# Prevention and management of health products shortages by the French national agency (ANSM), 10 years of experience

**DOI:** 10.3389/fpubh.2023.1293110

**Published:** 2023-11-17

**Authors:** Laëtitia Belgodère, Joseph Emmerich, Nicolas Albin, Trystan Bacon, Pascale Daynes, Stéphane Vignot, Thierry Vial, Guillaume Renaud, Carole Le Saulnier, Corine Maillard-Couvreur, Mélanie Cachet, Marie-Laure Veyries, Rym Youdarene, Wahiba Oualikene-Gonin, Christelle Ratignier-Carbonneil, Patrick Maison

**Affiliations:** ^1^Agence Nationale de Sécurité du Médicament et des Produits de Santé (ANSM), Saint Denis, France; ^2^Hôpital Saint Joseph Paris (Médecine Vasculaire), INSERM 1153 CRESS, Université de Paris Cité, Paris, France; ^3^Groupe Hospitalier Mutualiste de Grenoble, Institut Daniel Hollard, Grenoble, France; ^4^General Practitioner, Montmerle-sur-Saône, France; ^5^Collège universitaire de médecine générale, Université Claude Bernard Lyon 1, Lyon, France; ^6^Groupe de travail "Thérapeutique", Collège de la médecine générale, Paris, France; ^7^Union Francophone des Patients Partenaires, Faculté de Médecine, Centre Hospitalier Universitaire Grenoble Alpes, La Tronche, France; ^8^Institut Godinot, Reims, France; ^9^Hospices Civils de Lyon, Lyon, France; ^10^EA 7379, Epiderme, Faculté de Santé, Université Paris-Est Créteil, Créteil, France; ^11^CHI Créteil, Créteil, France

**Keywords:** shortage, regulatory science, medical device, drug, health policy

## Abstract

Shortages of drugs and medical devices have tended to increase in France and worldwide, with consequences for patients and healthcare professionals. Preventing shortages of health products has become a priority for regulatory authorities, including the French National Agency for Medicines and Health Products Safety (ANSM). To highlight perspectives for a better prevention, we described and analyzed the management of shortages in the availability of health products in France over the last 10 years. The supply chain was mapped to identify the main causes of shortages and stakeholders involved in managing shortages throughout the supply chain. National and European initiatives and regulatory measures were reviewed. A retrospective nationwide data analysis from the French reporting system of health product shortage reports was conducted over 10 years for drugs (2013–2022) and over an 18-month period for medical devices, from 1st March 2022 to 31st August 2023. An increase in drug shortage reports was observed, rising from 404 in 2013 to 3,761 in 2022 for drugs, with a relatively constant distribution of affected therapeutic classes. In 2022, the main reported causes of drug shortage risk were insufficient production capacity (27.1%), increased sales volume (21.5%), or lack of supply (13.6%). Over half of the reports on medical devices (55.4%) were objectified as indispensable, and their causes were mainly due to a lack of supply (48.2%), discontinuation of marketing (14.9%), increased sales volume (13.2%), and regulatory reasons (9.6%). ANSM and French authorities have engaged a public health policy for prevention and management of health product shortages including financial penalties, minimum safety stocks for Major Therapeutic Interest drugs, and a shortage management plan. Based on 10 years of experience, four priority measures have been identified to anticipate the risk of heath products shortages based: the importance of a national coordination from raw materials to local market, the implementation of new prevention and management actions in the supply chain, strengthening European cooperation and regulation including the establishment of a list of critical drugs, and promoting transparency and information.

## Introduction

Over the last decade, an increasing trend of drug and medical device shortages has been observed in countries all over the world, independently of their income levels or geographical area (1–4). Increasingly frequent, these health products shortages have a significant impact on patients, healthcare professionals, and the economy (5–10). Regulatory authorities may also allocate huge amounts of resources to manage health products shortages. Therefore, the prevention of shortages has become a priority for authorities worldwide (11–19). This global problem requires the implementation of specific measures to improve prevention, anticipation, and ensure better coverage of medical needs.

In the US, at the height of the drug shortage crisis, the number of new shortages quadrupled, rising from 61 shortages in 2005 to a peak of 251 in 2011 (4). The number of new drug shortages has been decreasing since 2011, reaching 49 in 2022 driven by preventive measures taken by many groups including the FDA (4).

In Europe, the number of drug shortages has grown 20-fold between 2000 and 2018, compromising European healthcare systems (20, 21). The COVID pandemic has highlighted the vulnerability of health products availability in a public health emergency situation (6, 22–24). European Medicine Agency’s (EMA), whose role has been strengthened by a new European regulation (25), took up the issue of managing health products shortages at the time of the health crisis with coordination actions (11, 16). France has been addressing this concern for several years. Various measures have recently been implemented at national level, including financial penalties, minimum safety stocks and shortage management plan for major therapeutic interest drugs (articles L5121-29 to L5121-34 of the Public Health Code) (13, 26).

In this context, the aim of this paper is to describe and analyze the reported data on shortages in the health products availability in France over the last ten years, and to highlight strategies for a better prevention.

## Analysis of the management of shortages in the availability of health products in France

### Mapping the risk of shortages within the health products supply chain

**Figure 1:**
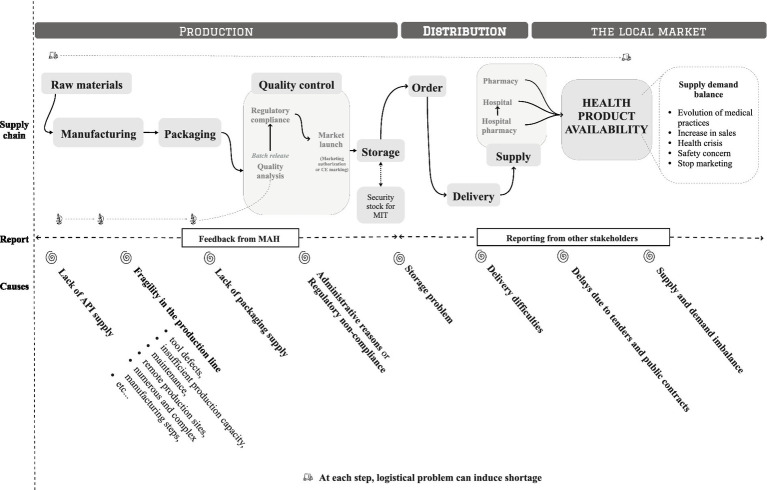
Supply chain and main causes of shortages in the availability of health products (drugs and medical devices).

Figure 1 depicts a mapping of the supply chain and identifies the main causes of shortages. The map was carried out, following stakeholder exchanges, using Miro software, version 0.7.37. This overview provides insight into the extent of action required and emphasizes the involvement of various stakeholders across the health products supply chain: industrials at the manufacturing level, regulators, and other stakeholders impacting the market.

The identified causes are categorized according to the different stages of the supply chain. These include weaknesses in the production chain, such as insufficient supply, manufacturing failures, and non-compliance with regulatory requirements. Distribution issues, such as storage or shipping problems, also contribute to shortages. Other factors, that may affect the health products market, include delays due to tenders and public contracts as well as imbalances between supply and demand. In addition, logistical difficulties may arise at any stage of the supply chain.

### Data from the French health products shortage notification system

Our observational retrospective study analyzed reports of health products shortages from the French national system.

In France, the Public Health Code defines a shortage as the impossibility of manufacturing or using a medicine (CSP Art. R. 5124-49-1). Pharmaceutical companies, which are responsible for ensuring the availability of the medicines they market in France, notify ANSM of the risk of stock shortage or actual stock shortages. Since 2012, manufacturers of major therapeutic interest drugs have been required to promptly notify the ANSM of any potential or actual supply chain shortages that could lead to or have resulted in a shortage or to face financial penalties (Decrees 2012-1096 of 28th September 2012 and 2016–993 of 20th July 2016). The reporting system database contains information reported by Marketing Authorization Holder (MAH) through completed declaration forms. The following data were analyzed over a ten-year period between 2013 and 2022: number of reports, type of declaration (shortage or shortage risk), and ATC codes of the major therapeutic interest drug concerned (according to the WHO Collaborating Centre for Drug Statistics Methodology) (27). The causes for major therapeutic interest drugs have been reported since 2021 from reports declared in the new e-portal (Trustmed), and they were analyzed over the year 2022.

For medical devices, reports of risks of shortages or disruptions in availability were received following the implementation of a non-mandatory procedure specific to medical devices established by ANSM, and analyzed over an 18-month period from 1^st^ March 2022 to 31^st^ August 2023. This procedure, for the shortages management by anticipation, is based on the principle of an early exchange of information.

The categories of causes were elaborated on the basis of a working group involving the various stakeholders. All available reports have been analyzed to describe the production phase. Data were presented as numbers or percentages of major therapeutic interest drugs or medical devices reported on shortage or risk shortage.

**Figure 2:**
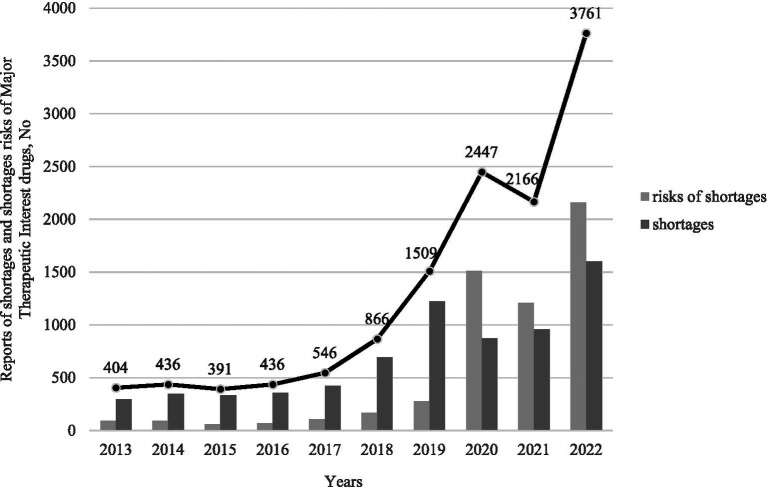
Number of reports of risks of shortages and shortages for major therapeutic interest drugs between 2013 and 2022 in France.

**Figure 3:**
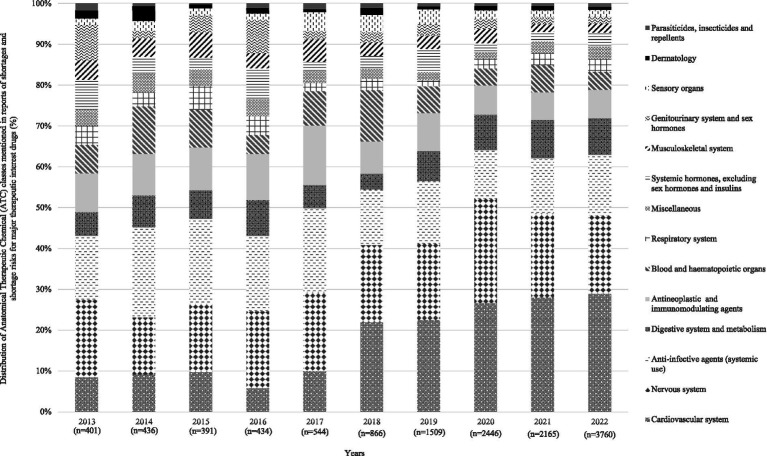
Distribution of Anatomical Therapeutic Chemical (ATC) classes mentioned in reports of shortages and shortage risks for major therapeutic interest drugs (2013–2022).

**Figure 4:**
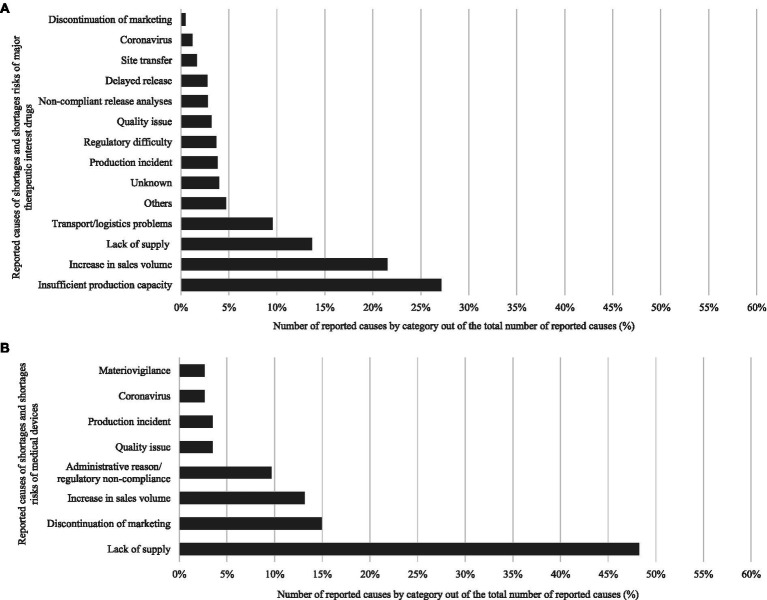
Percentages of reported causes of risks of shortages and shortages of major therapeutic interest drugs **(A)** and medical devices **(B)** in France.

Figure 2 displays the number of reports of shortage risk and shortage for major therapeutic interest drugs in France between 2013 and 2022 (from 404 to 3,761). Figure 3 indicates a relatively constant distribution of therapeutic classes affected by shortages over time with cardiovascular system, nervous system, and anti-infective drugs accounting for approximately 50% of reports. In 2022, 4,372 causes of shortage risk or shortages of major therapeutic interest drugs were reported, mainly due to insufficient production capacity and increased sales volume (27.1 and 21.5% respectively), and 13.6% due to a lack of supply (raw material and/or packaging material) (Figure 4A).

Although less impacted, medical devices are also concerned about shortages. Over an 18-month collection period in 2022–2023, 184 shortage risks and shortages in availability were reported in France. Among these, 102 were objectified as indispensable for the continuity of care. 52.9% were reported by manufacturers or distributors. Shortages in availability were mainly due to a lack of supply (48.2%), discontinuation of marketing (14.9%), increased sales volume (13.2%), and regulatory reasons (9.6%) (Figure 4B). Class IIa and III medical devices are more affected by shortages (37.1 and 28.9% respectively) than class I and IIb medical devices (15.5 and 17.5% respectively).

### Actionable recommendations

Based on our ten-year period and taking into account the mapping of stages, our results and the review of initiatives and regulatory measures taken since 2012, four priority measures have been identified to anticipate the risk of health products shortages, following the roadmaps of the French government (26) and the European Commission (28).

#### Importance of a national coordination

To date, the monitoring of the downstream supply chain is not complete and centralized (Figure 1). National coordination from raw materials to the local market allow to facilitate their management and anticipate shortage risks more effectively. To manage shortages and limit their impact, regulatory actions could be granted, such as allowing the importation of therapeutic alternatives, extending batch expiration dates, or authorizing hospital preparations (e.g., cisatracurium during the COVID crisis) (6).

Effective coordination requires the centralization of knowledge on issues related to all stages of the supply chain (Figure 1), including manufacturing data, logistics, needs, and use, but also agreements and contracts concluded by stakeholders. National governance can play a key role in coordinating decisions and actions needed at national and territorial levels for the day-to-day management of health products shortages.

Economic factors such as price, reimbursement, and monopoly as well as therapeutic recommendations, consumption, and off-label use are important determinants of the availability and the balance between production and needs.

#### Implementation of new prevention and management actions during the supply chain

Since September 2021, French regulations require the MAH to maintain a minimum safety stock of two months for major therapeutic interest drugs reserved for the national market to prevent shortages more effectively. Decree 2021–349 of 30th March 2021 allows the ANSM to adjust stocks if necessary. In 2022, the minimum safety stock was increased to four months for 422 drugs. In addition, MAHs are required to submit shortage management plan (SMP) to the ANSM which identifies risks in the supply chain and outlines measures to mitigate them. It describes therapeutic alternatives allowing the continued patient treatment under the best possible conditions and actions to be taken if the shortage cannot be avoided. Non-compliance with these national obligations is subject to financial penalties, with reinforced guidelines from 2022.

In addition, to ensure the supply of health products, the shortage prevention system needs to be strengthened. Actions should be taken to improve compliance with shortage management plans and the respect of security stocks for major therapeutic interest drugs. Regulatory authorities should be empowered to require manufacturers to take all necessary measures to reduce and manage the risks of shortages of all health products, including medical devices.

Although reports of medical devices shortages are currently limited, a legislative lever has just been introduced (articles 27 of law no 2023–171 of 9th March 2023). The decree will enable the implementation of reporting obligations for operators. A regulatory framework will be established to consolidate the definition of the risk situation for patients caused by the unavailability of medical devices.

French legislators point out that French pharmaceutical production has declined, leading to a loss of French health sovereignty (29). Maintaining or even relocating active pharmaceutical ingredients and health products manufacturers in France and Europe would help to limit supply chain difficulties, which have been identified as a major source of shortages. Actions should be taken to promote and improve the attractiveness of France and Europe. A map of active and potential production sites in Europe should be shared in order to identify points of vulnerability in the production chain. Discussions on joint procurement, in particular for essential vaccines or other drugs at the European level, will be pursued.

#### Strengthening European cooperation and regulation to better prevent health product shortages

A strategy to secure the supply of health products is being implemented at the European level (16, 23, 30–33).

To reinforce the EMA role in crisis preparedness and management for health products, a new regulation has been implemented in 2022 (25). This could also provide a framework for dealing with shortages more broadly. A comprehensive crisis management plan also covers medical devices, which have not been taken into account at EMA level until now. A network of contact points was created (SPOC), and European working groups were set-up: the CHESSMEN joint action (Coordination and Harmonization of the Existing Systems against Shortages of Medicines, European Network), Executive Steering Group on Shortages and Safety of Medicinal Products (MSSG), a European collaboration by the EMA (Task Force) in 2016, and EU4Health program. To strengthen Europe’s ability to prevent, detect, and rapidly respond to cross-border health emergencies, the European Commission has established a Health Emergency Response Authority (HERA). A draft reform of the European Commission’s pharmaceutical legislation is currently being prepared (28). This would introduce enhanced monitoring of drug shortages across the European Union, including the establishment of a list of critical drugs (31). The ANSM is actively participating in the work on this issue European level and in the drafting of the new European regulation.

The establishment of a database on drug supply within the EMA allows for the regulation and facilitation of data consolidation. With regard to medical devices, the ANSM supports common measures and positions, in particular with regard to the provisions adopted at national level.

The COVID crisis has demonstrated the importance and feasibility of solidarity within the European Union (34–37) to reduce competition between member states in fighting shortage situations.

#### Promoting transparency and information to prevent the consequences related to health products shortages and enhance confidence

To enhance transparency, stakeholder interactions and access to information on health products availability at all stages of the supply chain, efforts must be made to improve in accordance with trade secrets. At the European level, efforts have been made to publish and share information on shortages (38).

There is also a need to share information in real time on the availability of health products, from suppliers to pharmacists and physicians, as soon as a significant shortage is identified and confirmed.^1^

All stakeholders, including health professionals and patient representatives, have a key role in managing the crisis induced by health products shortages, and should be involved in the decision-making process. In particular, they are consulted to identify real needs in the field, to establish national recommendations, and, where appropriate, to prioritize treatment indications (39–41).

As part of its policy of data sharing and transparency, the ANSM has developed a digital application^2^ intended for the general public and healthcare professionals. This application provides access to the history of reports and annual analyses.

Public understanding of the context is necessary for the proper management of health products shortages and compliance with the measures put in place. Public education and awareness should be raised through transparent communication, information campaigns, and integrating patient decisions in the management of drug shortages, in order to improve their confidence in the healthcare system for positive outcomes.

The impact of health products shortages on the care pathway and the loss of opportunity for patients should be objectively assessed and more precisely defined, whether in terms of treatment delays, risk of reduced efficacy, occurrence of adverse effects, or medication errors (42). The multifactorial and proactive approach in France aims to minimize the impact of shortages on patients and health professionals. Our work highlights the need to monitor the targets set by the ANSM to address this public health priority, and therefore to strengthen or develop existing indicators.

## Discussion

To our knowledge, this is the first analysis of both drugs and medical devices availability shortage reports over a long period of time and at a national level. The mapping process highlighted the fact that the shortages occurred at the same stage in the supply chain, for both drug and medical device. Tensions over drug availability are much more frequently reported than for medical devices.

Our data confirm an increase in reports of concerns about the availability of major therapeutic interest drugs in France over time. Regulatory changes over the study period make it difficult to interpret the data and make comparisons over the years. The implementation of the anticipation policy in 2020 may have contributed to the more than fourfold increase in reports, from 866 in 2018 to 3,761 in 2022. To compare our data quantitatively and qualitatively, and more specifically the number of drug reports, we need to take into account changes in regulations. This also highlights the fact that regulations can have an impact on the reporting system.

Similar increases have also been observed in other European countries, including Finland, the Netherlands, and Germany (3, 12). The therapeutic classes most affected were generally the same across different countries, particularly the nervous system and cardiovascular system (3, 14, 15). More international information and data are becoming available on this topic, although they cover shorter periods of time (38). However, comparisons are difficult due to the lack of common criteria for defining shortages and criticality (1). Indeed, there is a lack of standardization in the definition of shortages between the different European member states (1). Even if a harmonized definition within Europe has been agreed in 2019 (11), each member state has its own notification system with different characteristics (43). Consequently, our results give only visibility on shortages and their causes inherent to the production stages. To date, there is no systematic monitoring of the downstream supply chain. As a result, our data on shortages do not include the distribution chain failure (non-delivery of a pharmacy or internal-use pharmacy), and supply disruptions (inability of a pharmacy to dispense a drug to a patient within 72 h).

The issue of shortages and their management in the field of medical devices is a recent concern. With a year and a half’s hindsight, this analysis is preliminary and there is little data available from other countries. As reporting on medical devices was not mandatory for the industrials, declarations provided from a variety of sources (patients, healthcare professionals, industrials…), whereas drug shortages are declared by the marketing authorization holders or the pharmaceutical companies operating them. Furthermore, reports from the professionals and patients have been essential so far, accounting for over a third of the reports. As a result, manufacturers’ and distributors’ statements are not exhaustive, as is probably the case with drugs. Moreover, quantitative comparisons between reports on shortages of medical devices and drugs are not straightforward, due to the difference in term of regulations and type of declarant.

The European level is a framework for exchange, with the aim of fostering cooperation, encouraging dialog and promoting harmonization and use of preventive practices to reduce shortages between member states. Management including legislation, price negotiations and market issues are at the national level. To extend the efforts made at the national level, ANSM is a driving force at the EMA level in the CHMP and in the working groups. In particular, the agency shares its experience of the national pilot phase for medical devices at EMA level, with the aim of strengthening regulatory measures to anticipate future developments.

Reporting and data analysis allow us to characterize shortages, adapt actions, and evaluate their impact. Comprehensive data on the entire chain and on the characteristics of drug production will make it possible to model and anticipate disruptions.

The fact that the study does not describe all factors that may affect the availability of health products may be a limitation. Price is often mentioned as a determining factor in dealing with shortages of health products. However, pharmaceutical pricing policies are not part of the European framework, but are the result of national decisions. Negotiations, managed at the national level, are a weakness of the European system.

This integrated approach includes: a well-coordinated approach and management in the face of a shortage, an improvement in communication and operations within all the stakeholders, changes in government policy (at the national and European level to achieve a harmonized policy); and by education and training of healthcare professionals but also of patients.

The implementation of such a strategy at the national level, targeting all different measures together, should hopefully reduce the impact of health products shortages. Similar measures to reduce the impact of shortages appear to have been successful in the US (4).

It should be kept in mind that the existing gaps between supply and demand are another critical parameter in the balance of the global health system and particularly in France where the consumption of health products is known to be high (44–48).

## Conclusion

Within the European framework, France has implemented a new mitigation strategy regarding drugs and medical devices supply chain tensions and shortages. The ANSM is involved in a broad public health policy to improve the prevention, management, and response to health products supply tensions and shortages, which is based on 4 priorities: coordination, prevention/management actions, European cooperation, and transparency. The aim of this strategy is to reduce the occurrence of health products shortages and the ensuing consequences for patients. The ANSM therefore supports all initiatives at national and European level in line with this strategy. Manufacturers, MAHs, hospitals, health-care professionals, patients, and regulators must all be involved to strive against shortages of drugs and medical devices.

## Author contributions

LB: Conceptualization, Formal analysis, Investigation, Methodology, Writing – original draft, Writing – review & editing. JE: Conceptualization, Writing – review & editing. NA: Writing – review & editing. TB: Writing – review & editing. PD: Writing – review & editing. SV: Writing – review & editing. TV: Writing – review & editing. GR: Writing – review & editing. CS: Writing – review & editing. CM-C: Writing – review & editing. MC: Writing – review & editing. M-LV: Writing – review & editing. RY: Writing – review & editing. WO-G: Writing – review & editing. CR-C: Writing – review & editing. PM: Validation, Writing – review & editing.

## Footnotes

^1^ https://ansm.sante.fr/disponibilites-des-produits-de-sante

^2^ https://data.ansm.sante.fr/
